# Tubeless versus standard percutaneous nephrolithotomy: an update meta-analysis

**DOI:** 10.1186/s12894-017-0295-2

**Published:** 2017-11-13

**Authors:** Yang Xun, Qing Wang, Henglong Hu, Yuchao Lu, Jiaqiao Zhang, Baolong Qin, Yudi Geng, Shaogang Wang

**Affiliations:** 1 Department of Urology, Tongji Hospital, Tongji Medical College, Huazhong University of Science and Technology, No.1095 Jiefang Avenue, Wuhan, China; 2 Reproductive medicine center, Tongji Hospital, Tongji Medical College, Huazhong University of Science and Technology, Wuhan, China

**Keywords:** Percutaneous nephrolithotomy, PCNL, Tubeless, Update, Meta-analysis

## Abstract

**Background:**

To update a previously published systematic review and meta-analysis on the efficacy and safety of tubeless percutaneous nephrolithotomy (PCNL).

**Methods:**

A systematic literature search of EMBASE, PubMed, Web of Science, and the Cochrane Library was performed to confirm relevant studies. The scientific literature was screened in accordance with the predetermined inclusion and exclusion criteria. After quality assessment and data extraction from the eligible studies, a meta-analysis was conducted using Stata SE 12.0.

**Results:**

Fourteen randomized controlled trials (RCTs) involving 1148 patients were included. Combined results demonstrated that tubeless PCNL was significantly associated with shorter operative time (weighted mean difference [WMD], −3.79 min; 95% confidence interval [CI], −6.73 to −0.85; *P* = 0.012; I^2^ = 53.8%), shorter hospital stay (WMD, −1.27 days; 95% CI, −1.65 to −0.90; *P* < 0.001; I^2^ = 98.7%), faster time to return to normal activity (WMD, −4.24 days; 95% CI, −5.76 to −2.71; P < 0.001; I^2^ = 97.5%), lower postoperative pain scores (WMD, −16.55 mm; 95% CI, −21.60 to −11.50; P < 0.001; I^2^ = 95.7%), less postoperative analgesia requirements (standard mean difference, −1.09 mg; 95% CI, −1.35 to −0.84; P < 0.001; I^2^ = 46.8%), and lower urine leakage (Relative risk [RR], 0.30; 95% CI 0.15 to 0.59; *P* = 0.001; I^2^ = 41.2%). There were no significant differences in postoperative hemoglobin reduction (WMD, −0.02 g/dL; 95% CI, −0.04 to 0.01; *P* = 0.172; I^2^ = 41.5%), stone-free rate (RR, 1.01; 95% CI, 0.97 to 1.05; *P* = 0.776; I^2^ = 0.0%), postoperative fever rate (RR, 1.05; 95% CI, 0.57 to 1.93; *P* = 0.867; I^2^ = 0.0%), or blood transfusion rate (RR, 0.79; 95% CI, 0.36 to 1.70; *P* = 0.538; I^2^ = 0.0%). The results of subgroup analysis were consistent with the overall findings. The sensitivity analysis indicated that most results remained constant when total tubeless or partial tubeless or mini-PCNL studies were excluded respectively.

**Conclusions:**

Tubeless PCNL is an available and safe option in carefully evaluated and selected patients. It is significantly associated with the advantages of shorter hospital stay, shorter time to return to normal activity, lower postoperative pain scores, less analgesia requirement, and reduced urine leakage.

## Background

As a common urological disease, the prevalence rates for urinary stones vary from 1% to 20%. In countries with a high standard of life such as Canada or the United States of America (USA), renal stone prevalence is notably high (>10%) [1]. Urinary stones can cause renal function injury, which has a great impact on public health. With the advances of surgical technology, less invasive procedures such as percutaneous nephrolithotomy (PCNL) have gradually become a preferred therapy for urinary stone in the last two decades [2, 3]. Using a nephrostomy tube for drainage has been considered the standard procedure after PCNL [4]. Since Bellman first introduced tubeless PCNL in 1997 [5], the interest and enthusiasm of this surgical procedure had been widespread. PCNL without postoperative nephrostomy tube placement is defined as tubeless PCNL. When neither a nephrostomy tube nor a ureteral stent is used, the procedure is commonly regarded as total tubeless PCNL [6]. A large number of studies on tubeless PCNL have been performed and several previously published systematic reviews have reported its efficacy and safety [4, 7–9]. However, the available evidence is still currently inconclusive because of the limited quality and quantity of the analyzed randomized controlled trials (RCTs). More rigorously designed RCTs are required to collect better evidence to support the use of tubeless PCNL. Furthermore, tubeless PCNL has not been generally accepted in clinical medicine, probably due to concerns of urine leakage, obstruction by residual stone fragments, or requirement for repeat access [10]. Since 1997, several RCTs [11–24] have been performed to compare the safety and effectiveness of tubeless and standard PCNL, including five high-quality RCTs published after 2012 [20–24]. These latest publications need to be included in an updated review to explore the most recent evidence on the use of tubeless PCNL. Therefore, we performed a meta-analysis to update previously published systematic reviews on the efficacy and safety of tubeless PCNL.

## Methods

### Literature search

We performed a systematic literature search of Medline (using PubMed as the search engine), Web of Science databases, EMBASE (using Ovid as the search engine) and the Cochrane Library to confirm relevant studies in accordance with Cochrane standards, and PRISMA (Preferred Reporting Items for Systematic Reviews and Meta-Analyses) guidelines [25] in November 2016 and updated in March 2017. There were no strict restrictions of year or language in the searching process. The search was performed with the following terms in combination to identify relevant studies: (“total tubeless” or “tubeless”) and (“percutaneous nephrolithotomy” or “percutaneous lithotripsy” or “PCNL” or “PNL” or “PCN”). Two authors screened all the citations and abstracts independently. All potentially eligible studies involving comparison of the tubeless and standard PCNL were included.

### Selection criteria

Inclusion criteria were: (1) RCTs; (2) studies published in English; (3) studies comparing tubeless and standard PCNL; (4) patients included in the studies were suited to PCNL, with no ureteric obstruction, no significant bleeding during the surgery, and no major collecting-system injury or normal renal function; (5) the nephrostomy tube placed at the completion of the procedure in the standard PCNL group; (6) patients included in the tubeless PCNL group were contraindicated from using a nephrostomy tube, however those with a double-J stent or external ureteral catheter could be considered; (7) studies reported at least one of the following clinical outcomes: operative time, hospital stay, postoperative hemoglobin drop, postoperative analgesic requirement, return to normal activity, postoperative pain score, stone free rate, or major complications.

The exclusion criteria included: (1) pediatric patients under 14 years of age; (2) non-RCTs; (3) patients who underwent bilateral simultaneous PCNL; (4) patients with staghorn stones, congenital urinary tract anomalies, serious urinary infection, solitary functioning kidneys, or kidneys with prior open surgery.

Two reviewers completed the selection process independently.

### Data extraction

Data extraction and quality evaluation were carried out by two reviewers. The information including study name, authors, publication year, country, study design, interventions, size of the drainage tube, number of patients, age, gender, stone burden, and clinical outcomes of interest (stone-free rate, operative time, hospital stay, return to normal activity, postoperative hemoglobin drop, postoperative analgesic requirements, postoperative pain score (VAS), blood transfusion, fever, urine leakage) were extracted from each included study. With the purpose of reducing the heterogeneity of the different studies and to make them easier to describe and understand, operative time was reported in minutes as a unit for all studies. Pain score was transformed by using linear 100 mm (the range of 0–100) visual analogue scale (VAS) (0 no pain, 100 maximum intolerable pain) uniformly [26]. In order to perform the sensitivity analysis, the type of drainage was also extracted from each study.

### Assessment of quality

The criteria provided by the Oxford Center for Evidence-Based Medicine [27] was used to assess the level of evidence for all studies. The risk of bias for each RCT included was evaluated by two reviewers independently, according to the Cochrane Collaboration’s tool [28]. The tool includes six aspects: random sequence generation, allocation concealment, blinding of participants and personnel, blinding of outcome assessment, incomplete outcome data, selective reporting, and other biases. The risk of bias was analyzed via the Cochrane Review Manager (REVMAN 5.3). Disagreements were resolved by discussion.

### Statistical analysis

A meta-analysis was conducted to compare the effectiveness and safety of tubeless PCNL with standard PCNL. All statistical analyses were performed using Stata software (Stata SE 12.0). We chose statistical analysis methods and size effects based on data types and evaluation purposes. Relative risk (RR) was used for dichotomous data, while continuous data was evaluated using weighted mean difference (WMD) or standardized mean difference (SMD). If continuous data was presented as means and range, the methodology described by Hozo et al. was used for calculating the standard deviations (SD) [29]. All the results were reported with 95% confidence intervals (95% CI). The heterogeneity of included studies were evaluated via the chi-square test and quantified by measuring I^2^ value. When I^2^ values did not exceeded 50%, the fixed-effect model was used to calculate pooled estimates. However, if an I^2^ value exceeded 50% and could not be resolved by subgroup analysis, which indicated significant heterogeneity among the included studies, the random-effect model was used. The Z-test was used to determine the pooled effects, and *P*-value <0.05 was considered to be statistically significant. Forest plots were carried out to express the results of the meta-analysis. Influence analysis was performed to evaluate the effect of the included studies on the inter-study heterogeneity. The publication bias was assessed by Begg’s test. Furthermore, sensitivity analysis was carried out to detect the influence of the total tubeless, partial tubeless, and standard PCNL studies on the overall effect.

## Results

### Study characteristics

**Fig. 1:**
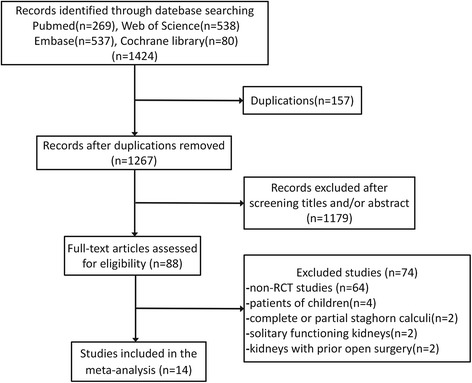
Flowchart of the literature search and studies selection. RCT = randomized controlled trial

Based on the inclusion and exclusion criteria, 14 RCTs [11–24] were finally included in our meta-analysis, and consisted of 576 patients who underwent tubeless PCNL and 572 patients who underwent standard PCNL. The process used for literature search and study selection is shown in Fig. 1. The studies included five RCTs [16–19, 24] which compared total tubeless PCNL with standard PCNL and nine RCTs [11–15, 20–23] which discussed partial tubeless PCNL. Table 1 shows the basic characteristics of the studies. The characteristics of the stones, including stone burden, stone location, and stone composition are summarized in Table 2. Age, sex ratio, and all stone characteristics described were comparable for the tubeless and standard PCNL groups in each study. The sex ratio was not reported in one study [11] and three studies [13, 18, 22] did not mention the size of the drainage tube. Other baseline characteristics which may have affected the outcome measures, such as body mass Index (BMI) [11, 13, 18, 19, 23], stone side [16, 19, 23, 24], number of punctures [10, 12, 22, 23], subcostal and supracostal punctures [13, 14, 17], and auxiliary procedures [13, 14, 18, 19, 21, 22, 24] were also reported in some studies. All data was also comparable between the two groups for each study.

**Table 1:** The basic characteristics and methodological quality of included studies

Study	year	nation	study design	Level of evidence	PCNL (tubeless/standard)	Drainage (Size of tube)	Sample size	Age(Year)	Sex (M/F)	Outcome measures
Kumar	2016	India	RCT	Level 2	Total tubeless	–	56	36.20 ± 13.32	33/23	1, 2, 3, 4, 5,
					Nephrostomy tube	18F	57	36.00 ± 11.82	31/26	6, 7, 8, 9, 10
Sebaey	2016	Egypt	RCT	Level 2	External ureteric catheter	6F	40	40.6 ± 11.9	31/9	1, 2, 3,
					Nephrostomy tube	14F	40	46.1 ± 18.4	27/13	5, 6, 10
Zhao	2016	American	RCT	Level 2	Double-J ureteral stent	–	15	54.7 ± 12.2	9/6	1, 2, 3, 5,
					Nephrostomy tube	8F/10F	15	58.3 ± 17.6	8/7	6, 8, 9
Agrawal	2014	India	RCT	Level 2	Double-J ureteral stent	5F/26cm	83	33 (18–55)	62/21	1, 3, 4, 5,
					Nephrostomy tube	12F	83	31 (21–57)	59/24	6, 7, 8, 9, 10
Lu	2013	China	RCT	Level 2	External ureteric catheter	F5	16	43.81 ± 18.89	6/10	1, 2, 3,
					Nephrostomy tube+ Double-J ureteral stent	F16 + F6	16	46.25 ± 22.37	7/9	5, 9, 10
Chang	2011	Taiwan	RCT	Level 2	Total tubeless	–	68	59.22 ± 12.44	51/17	1, 2, 3, 4,
					Nephrostomy tube + Double-J ureteral stent	F20 + F7	63	58.70 ± 10.85	50/13	5, 6, 7, 10
Aghamir	2011	Iran	RCT	Level 2	Total tubeless	–	35	38.4 ± 11.7	23/12	1, 2, 3, 4,
					Nephrostomy tube + Double-J ureteral stent	NA	35	40 ± 11.95	21/14	5, 6, 8
Istanbulluoglu	2009	Turkey	RCT	Level 2	Total tubeless	–	45	47.48 ± 13.04	25/20	1, 2, 3, 5,
					Nephrostomy tube	14F	45	43.91 ± 14.44	24/21	6, 8, 9, 10
Crook	2008	The United Kingdom	RCT	Level 2	Total tubeless	–	25	53	15/10	1, 3, 5, 6, 8
					Nephrostomy tube	26F	25	52	19/6	
Singh	2008	India	RCT	Level 2	Double-J ureteral stent	NA	30	31 (14–55)	14/16	1, 2, 3, 4,
					Nephrostomy tube	22F	30	34 (17–55)	15/15	5, 6, 7, 8, 10
Shah	2008	India	RCT	Level 2	Double-J ureteral stent	6F	33	44.18 ± 13.13	20/13	1, 2, 3, 5,
					Nephrostomy tube	8F	32	46.69 ± 12.46	21/11	6, 7, 8, 9, 10
Agrawal, Madhu S	2008	India	RCT	Level 2	Double-J ureteral stent	6F/26cm	101	33 (18–55)	76/25	1, 3, 4, 5,
					Nephrostomy tube	16F	101	31 (21–57)	76/25	6, 7, 8, 9, 10
Tefekli	2007	Turkey	RCT	Level 2	External ureteric catheter	5F	17	38.4 ± 12.3	8/9	1, 2, 3,
					Nephrostomy tube	14-F	18	41.3 ± 14.7	11/7	5, 6
Choi	2006	American	RCT	Level 2	Double-J ureteral stent	6F	12	52.9 ± 14	NA	2, 3, 4,
					Nephrostomy tube	8.2F	12	47 ± 16	NA	6, 7, 8

*RCT* Randomized Controlled Trial, *PCNL* Percutaneous Nephrolithotomy, *NA* Not Available (Insufficient Information Provided)

1, Stone-free rate; 2, Operative time; 3, Hospital stay; 4, Return to normal activity; 5, Postoperative hemoglobin drop; 6, Postoperative analgesic requirements; 7, Postoperative pain scores; 8, Blood transfusion; 9, Fever; 10, Urine leakage

**Table 2:** The basic characteristics of stones

Study	PCNL (tubeless/standard)	Stone burden	Stone location	Stone composition
Kumar	TB	30.2 ± 4.6 mm	Pelvic (35) Calyceal (15) Pelvic + Calyceal (6)	NA
	SD	29.5 ± 4.2 mm	Pelvic (20) Calyceal (24) Pelvic + Calyceal (13)	NA
Sebaey	TB	1.82 ± 0.36 cm	Renal pelvis (9) Lower calyx (25) Middle calyx (5) Upper calyx (1)	NA
	SD	1.91 ± 0.37 cm	Renal pelvis (8) Lower calyx (25) Middle calyx (5) Upper calyx (2)	NA
Zhao	TB	259.0 mm^2^	NA	Primarily calcium oxalate (53.3%) Mixed calcium (26.7%) Primarily uric acid (20%) Struvite (0%)
	SD	276.6 mm^2^	NA	Primarily calcium oxalate (40%) Mixed calcium (20%) Primarily uric acid (33.3%) Struvite (6.7%)
Agrawal	TB	3.8 (1.0–5.7) cm^2^	NA	NA
	SD	3.6 (1.1–5.3) cm^2^	NA	NA
Lu	TB	3.11 ± 0.62 cm	NA	NA
	SD	3.29 ± 0.54 cm	NA	NA
Chang	TB	Length 24.74 ± 2.69 mm Width 16.40 ± 3.65 mm	NA	Struvite + apatite (8–11.8%) Uric acid (0%) Whewellite (30–44.1%) Whewellite + apatite (13–19.1%) Weddellite (17–25%)
	SD	Length 24.86 ± 2.78 mm Width 15.29 ± 4.50 mm	NA	Struvite + apatite (7.9%) Uric acid (3.2%) Whewellite (38.1%) Whewellite + apatite (30.2%) Weddellite (20.6%)
Aghamir	TB	2.81 ± 0.59 cm^2^	NA	NA
	SD	2.87 ± 0.62 cm^2^	NA	NA
Istanbulluoglu	TB	448.93 ± 249.13 mm^2^	lower calyx (26) pelvis (11) multiple calyces(6) upper ureter (1) upper calyx (1)	NA
	SD	453.35 ± 165.97 mm^2^	lower calyx (14) pelvis (19) multiple calyces (10) upper ureter (2)	NA
Crook	TB	17.5 mm	NA	NA
	SD	21.6 mm	NA	NA
Singh	TB	750 mm^2^	Superior (9) middle (12) inferior calices (9)	mixture of calcium oxalate monohydrate /calcium oxalate dihydrate stones (71%) calcium phosphate (7%) carbonate apatite (7%) magnesium ammonium phosphate hexahydrate (7%) pure calcium oxalate monohydrate (7%) xanthine (1%)
	SD	800 mm^2^	Superior (9) middle (15) inferior calices (7)	
Shah	TB	535.36 ± 543.39 mm^2^	NA	Calcium oxalate monohydrate (60.6%) Calcium oxalate dihydrate (18.18%) Calcium phosphate (3.03%) Struvite (12.12%) Uric acid (6.06%) Cystine (0%)
	SD	495.91 ± 445.92 mm^2^	NA	Calcium oxalate monohydrate (53.12%) Calcium oxalate dihydrate (28.12%) Calcium phosphate (0%) Struvite (9.37%) Uric acid (6.25%) Cystine (3.12%)
Agrawal, Madhu S	TB	3.8 (1–5.7) cm^2^	NA	calcium oxalate (87%) struvite (7%) uric acid (1%)
	SD	3.6 (1.1–5.3) cm^2^	NA	calcium oxalate (84%) struvite (9%) uric acid (3%)
Tefekli	TB	3.0 ± 0.7 cm^2^	Renal pelvis (6) Lower pole calyx (11)	NA
	SD	3.1 ± 0.9 cm^2^	Renal pelvis (9) Lower pole calyx (9)	NA
Choi	TB	28.5 ± 15.4 mm	NA	NA
	SD	26.8 ± 13.5 mm	NA	NA

*TB* Tubeless Percutaneous Nephrolithotomy, *SD* Standard Percutaneous Nephrolithotomy, *NA*, Not Available (Insufficient Information Provided)

### Quality assessment

**Fig. 2:**
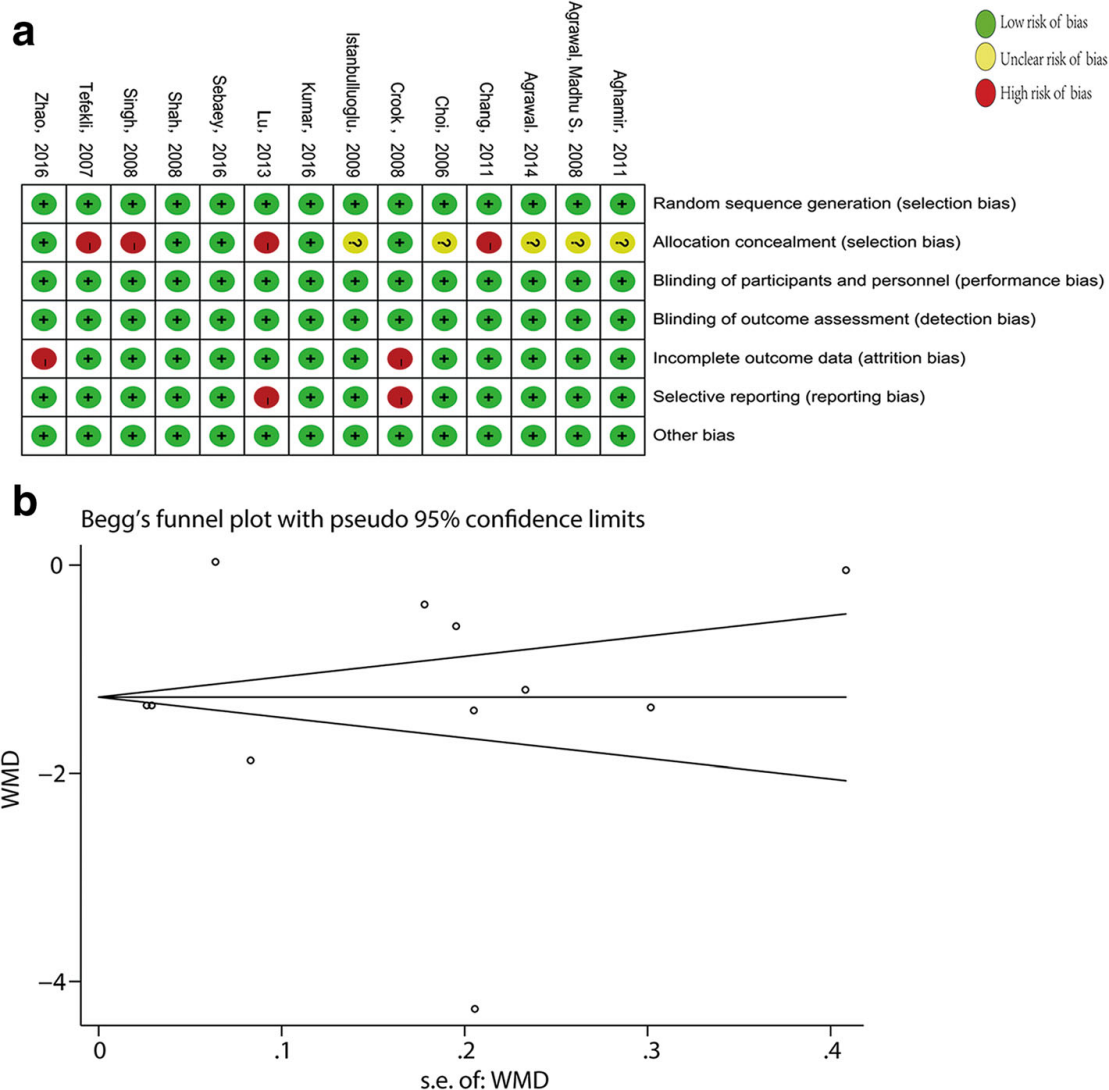
(**a**) Risk of bias for each included study. (**b**) Begg’ s funnel plot of the hospital stay. WMD = weighted mean difference

Table 1 shows that the level of evidence for each included study was graded Level 2. Fig 2A generalizes the results relative to the risk of bias for each randomized controlled study. As for the allocation concealment, five studies [11, 15, 17, 18, 21] did not describe the concealed method. Four studies [12, 13, 19, 20] were considered to have high risk of bias resulting from of the use of a random numbers table. There was a high risk of attrition bias in two studies [16, 22] due to the absence of SD. Two studies [16, 20] had a high risk of selective reporting bias because detailed explanation for some important outcomes was lacking.

### Publication bias

The publication bias was evaluated via Begg’s test. The Begg’s funnel plot of the hospital stay which was included in almost all studies is shown in Fig. 2B. The results of the Begg’s test for other factors are summarized in Table 3. Only postoperative pain score appeared to have a publication bias.

**Table 3:** Begg’s test for various factors

Factors	No. of studies	P value^a^	95% CI
Stone-free rate	11	0.956	[−0.711, 0 .675]
Operative time	9	0.385	[−3.073, 1.344]
Hospital stay	11	0.874	[−8.048, 9.303]
Return to normal activity	7	0.24	[−17.820, 5.653]
Postoperative hemoglobin drop	10	0.694	[−1.720, 1.202]
Postoperative analgesia equivalents	5	0.747	[−9.673, 12.096]
Postoperative pain scores	10	0.011	[1.664, 9.500]
Blood transfusion	7	0.545	[−2.640, 4.426]
Fever	7	0.627	[−1.311, 1.972]
Urine leakage	7	0.487	[−4.716, 2.585]

*CI* Confidence interval;

^a^P < 0.05 was considered statistically significant

### Influence analysis

**Fig. 3:**
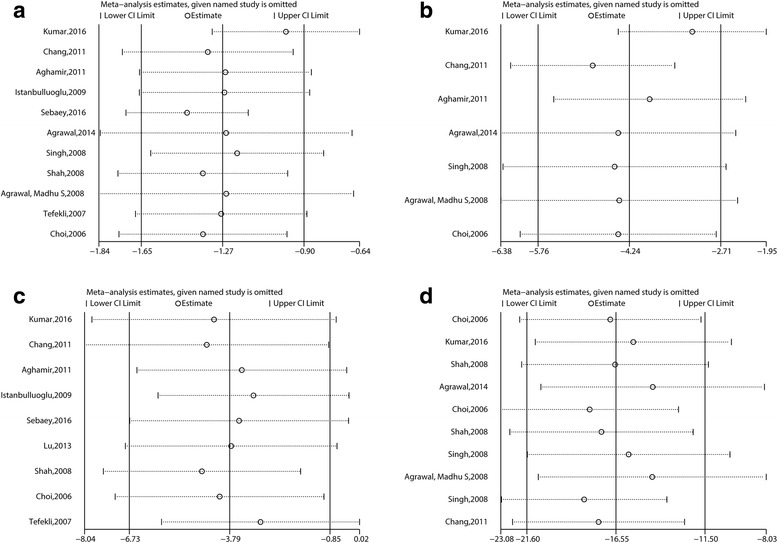
Influence analysis of (**a**) hospital stay, (**b**) return to normal activity, (**c**) operative time and (**d**) postoperative pain scores

For research indexes in which the I^2^ value exceeded 50%, such as the operative time, hospital stay, return to activities, and postoperative pain scores (VAS), influence analysis has been assessed to screen studies which have a significant effect on heterogeneity. The results are shown in Fig. 3.

### Meta-analysis

#### Operative time

**Fig. 4:**
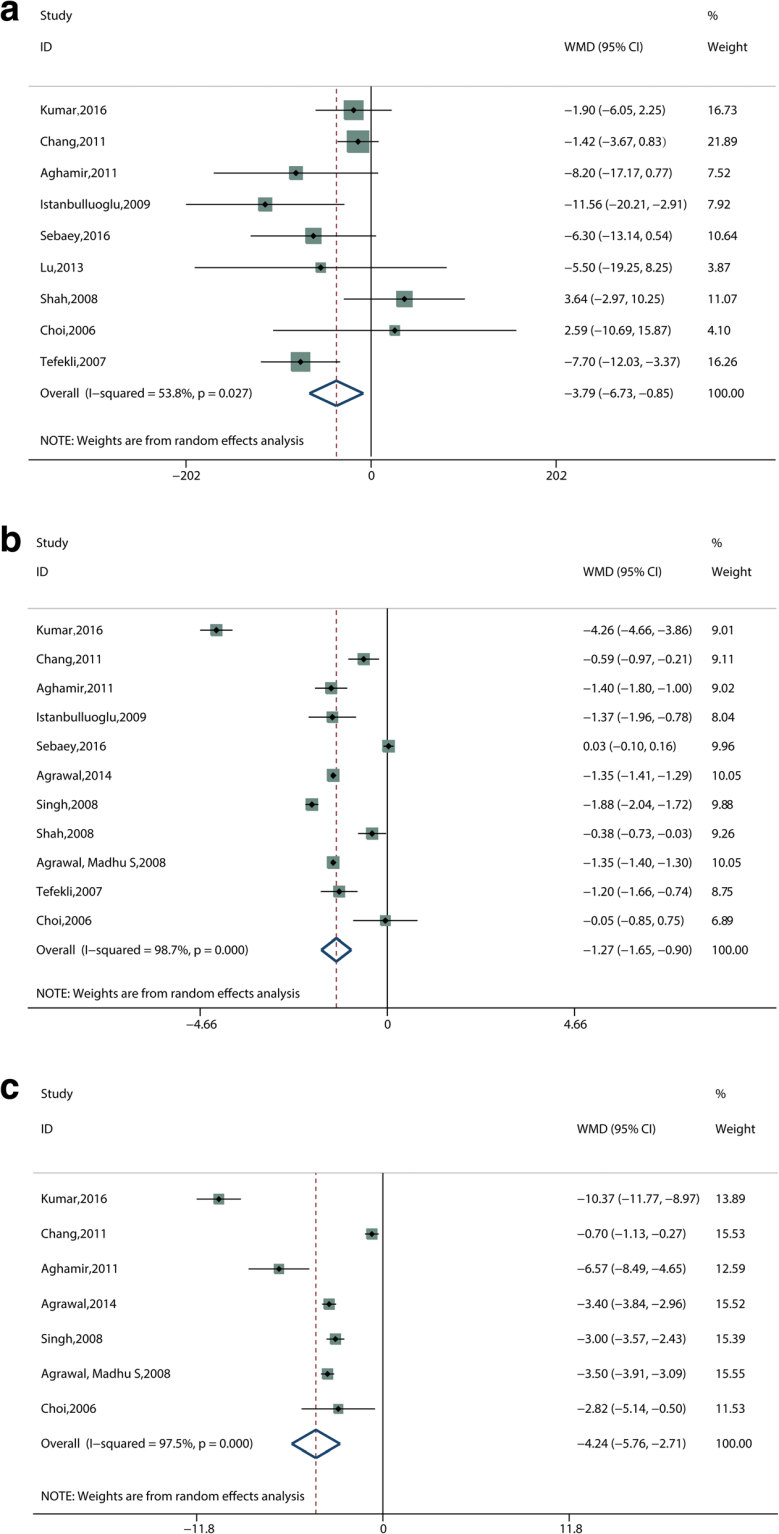
Forest plots and meta-analysis of (**a**) operative time, (**b**) hospital stay, (**c**) return to normal activity. WMD = weighted mean difference, CI = confidence interval

Eleven studies [11–14, 17–20, 22–24] assessed the operative time. Due to the lack of SD reported, two studies [13, 22] were excluded from the data combination. Analysis of the final nine studies showed that tubeless PCNL required less operative time than standard PCNL with a statistically significant difference (WMD, −3.79 min; 95% CI, −6.73 to −0.85; *P* = 0.012; I^2^ = 53.8%) (Fig. 4A).

#### Hospital stay

Hospital stay was measured in 14 studies [11–24]. Of these, 11 studies [11–15, 17–19, 21, 23, 24] were analyzed except for three trials [16, 20, 22], which did not report SD values. The pooled result showed the tubeless PCNL group was charged with a shorter hospital stay than the standard PCNL group (WMD, −1.27 days; 95% CI, −1.65 to −0.90; *P* < 0.001; I^2^ = 98.7%) (Fig. 4B).

#### Return to normal activity

Seven studies [11, 13, 15, 18, 19, 21, 24] assessed the time to return to normal activity between two groups. Meta-analysis of these studies via a random effect model showed that the tubeless PCNL group required a shorter time to return to normal activity than the standard PCNL group (WMD, −4.24 days; 95% CI, −5.76 to −2.71; P < 0.001; I^2^ = 97.5%) (Fig. 4C).

#### Postoperative hemoglobin drop

**Fig. 5:**
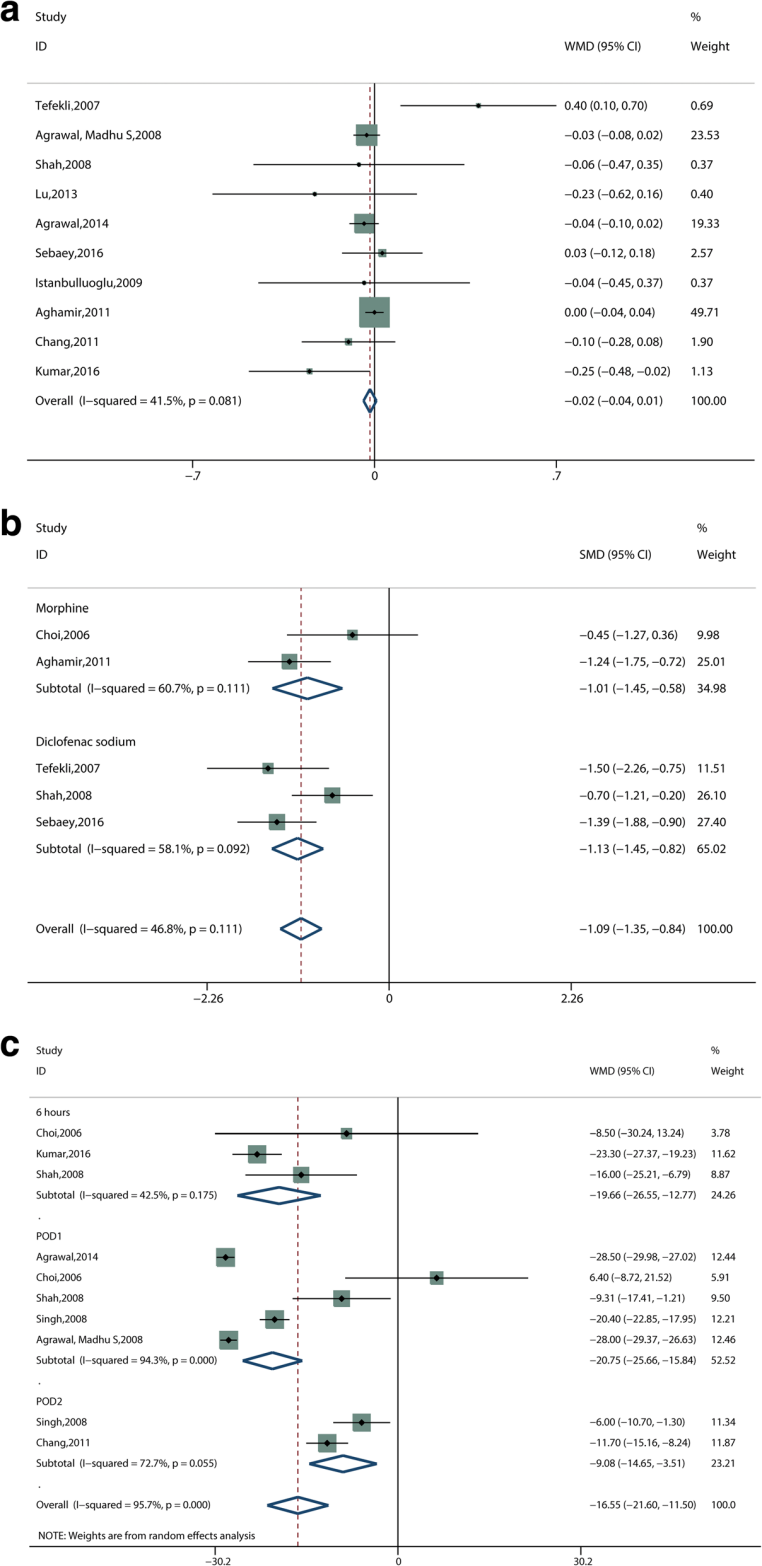
Forest plots and meta-analysis of (**a**) postoperative hemoglobin drop, (**b**) postoperative analgesia requirements, (**c**) postoperative pain scores. WMD = weighted mean difference, SMD = standardized mean difference, CI = confidence interval, POD = postoperative day

The mean (SD) estimated blood loss, reported by Choi et al. [11], was 72.73 (52.71) mL and 105 (68) mL in the tubeless PCNL group and the standard PCNL group, respectively. No statistical differences were found between the two groups (*P* > 0.05). Another 13 studies reported postoperative hemoglobin drop. Because the SD was not reported, three trials [13, 16, 22] were excluded from the data combination. A meta-analysis was performed for the other ten studies [12, 14, 15, 17–21, 23, 24], using a fixed effect model. The pooled results revealed no significant statistical differences between the tubeless group and standard PCNL group (WMD, −0.02 g/dL; 95% CI, −0.04 to 0.01; *P* = 0.172; I^2^ = 41.5%) (Fig. 5A).

#### Postoperative analgesia requirements

The study by Agrawal, Madhus et al. [15] showed the mean (SD) postoperative analgesic requirements (meperidine) in the first 24 h postoperatively was 81.7 (24.5) mg in the tubeless PCNL group and 126.5 (33.3) mg in the standard group. There was no significant statistical difference between the two groups (*P* < 0.01). Chang et al. [19] reported the postoperative analgesic requirements of both ketorolac and buprenorphine; the mean (SD) ketorolac dosage was 63.38 (15.22) mg in the tubeless PCNL group and 75.56 (16.44) mg in the standard PCNL group; and the dosage of buprenorphine was 0.09 (0.16) mg and 0.24 (0.76) mg in tubeless PCNL group and standard PCNL group, respectively. Ketorolac requirements favored the tubeless group (*P* < 0.001), but not the buprenorphine dosage (*P* = 0.09). The average tramadol dose required for analgesia postoperatively in Agrawal’s study [21] was 81.3 (24.3) mg and 128.0 (33.4) mg in the tubeless PCNL group and in the standard group, respectively; differences in doses were statistically significant (*P* < 0.001). As Kumar et al. [24] reported, cases in the tubeless group consumed significantly lower mean (SD) amounts of rescue analgesics (paracetamol), 1.48 (0.50) g in the tubeless group vs. 4.09 (1.11) g in the standard group (*P* < 0.05). A meta-analysis was also performed of the other five studies [11, 12, 14, 18, 23], using SMD for statistical analysis. These studies were divided into two subgroups according to the analgesic requirements (morphine or diclofenac sodium). The pooled result for the overall effect indicated the analgesic requirements in the tubeless PCNL group were significantly reduced (SMD, −1.09 mg; 95% CI, −1.35 to −0.84; *P* < 0.001; I^2^ = 46.8%) (Fig. 5B). In subgroup analysis, the results for each subgroup [“morphine” subgroup [11, 18] (SMD, −1.01 mg; 95% CI, −1.45 to −0.58; P < 0.001; I^2^ = 60.7%) and “diclofenac sodium” subgroup [12, 14, 23] (SMD, −1.13 mg; 95% CI, −1.45 to −0.82; P < 0.001; I^2^ = 58.1%) (Fig. 5B)], were consistent with the overall results.

#### Postoperative pain scores

Seven studies [11, 13–15, 19, 21, 24] reported postoperative pain scores estimated using the visual analog scale (VAS). Analyzing these studies revealed that the tubeless PCNL group had statistically significant lower postoperative pain scores (WMD, −16.55 mm; 95% CI, −21.60 to −11.50; P < 0.001; I^2^ = 95.7%) (Fig. 5C). Moreover, the results of the subgroup analysis for the “6 h” subgroup [11, 14, 24] (WMD, −19.66 mm; 95% CI, −26.55 to −12.77; P < 0.001; I^2^ = 42.5%), “POD1” subgroup [11, 13–15, 21] (WMD, −20.75 mm; 95% CI, −25.66 to −15.84; P < 0.001; I^2^ = 94.3%) and the “POD2” subgroup [13, 19] (WMD, −9.08 mm; 95% CI, −14.65 to −3.51; *P* = 0.001; I^2^ = 72.7%) (Fig. 5C), indicated the same tendency.

#### Stone-free rate

**Fig. 6:**
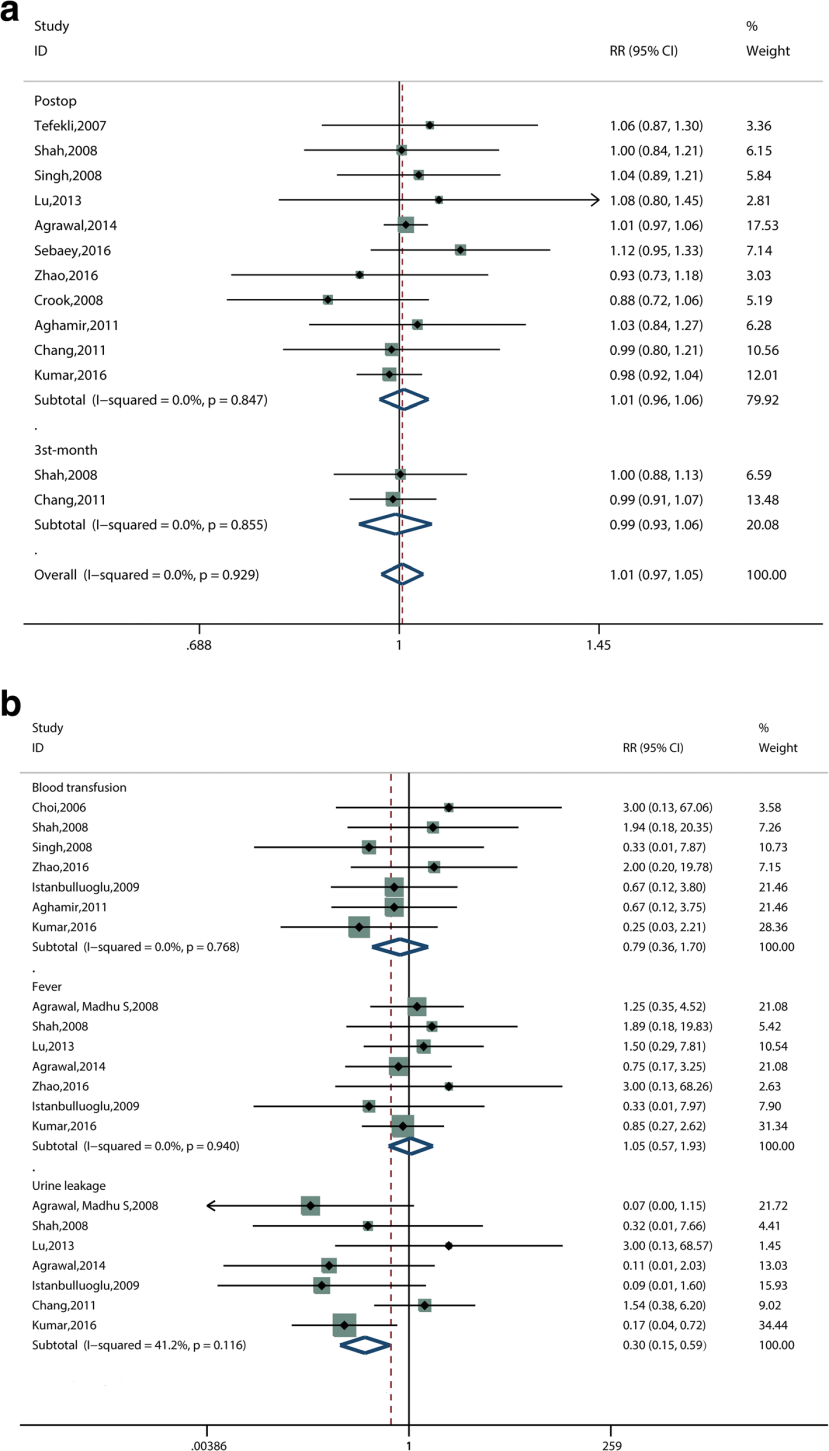
Forest plots of (**a**) stone-free rate, (**b**) blood transfusion, fever and urine leakage. RR = relative risk, CI = confidence interval

Eleven studies [12–14, 16, 18–24] reported the stone-free rate. The pooled results of the overall data showed no significant statistical difference between the tubeless PCNL and the standard PCNL groups (RR, 1.01; 95% CI, 0.97 to 1.05; *P* = 0.776; I^2^ = 0.0%) (Fig. 6A). The studies were divided into two subgroups according to the time when the stone-free rate was assessed. The pooled results of “the postoperative stone-free rate” subgroup, which included 11 studies [12–14, 16, 18–24], showed no significant difference between the two groups (RR, 1.01; 95% CI, 0.96 to 1.06; *P* = 0.703; I^2^ = 0.0%) (Fig. 6A). “The 3rd-month stone-free rate” subgroup consisted of two studies [14, 19] with similar results (RR, 0.99; 95% CI, 0.93 to 1.06; *P* = 0.834; I^2^ = 0.0%) (Fig. 6A).

#### Blood transfusion

The postoperative blood transfusion rate was measured in seven studies [11, 13, 14, 17, 18, 22, 24]. There was no significant statistical difference between the tubeless PCNL and the standard PCNL groups (RR, 0.79; 95% CI, 0.36 to 1.70; *P* = 0.538; I^2^ = 0.0%) (Fig. 6B).

#### Fever

Postoperative fever rate (temperature more than 38 °C) was evaluated in seven studies [14, 15, 17, 20–22, 24]. The meta-analysis of these studies revealed no statistical difference between the tubeless PCNL and the standard PCNL group (RR, 1.05; 95% CI, 0.57 to 1.93; *P* = 0.867; I^2^ = 0.0%) (Fig. 6B).

#### Urine leakage

Postoperative urine leakage was reported in seven studies [14, 15, 17, 19–21, 24]; a meta-analysis including these studies was performed using RR for effect size. Patients who underwent tubeless PCNL, rather than standard PCNL, were associated with a lower risk of postoperative urine leakage (RR, 0.30; 95% CI, 0.15 to 0.59; *P* = 0.001; I^2^ = 41.2%) (Fig. 6B).

#### Readmission

Several studies [12, 15, 24] mentioned the readmission rate, but in two of these studies, postoperative issues did not require readmission [12, 15]. Only Kumar et al. [24] reported that the standard PCNL group had a relatively higher rate of readmission, however the difference was not statistically significant between the two groups (7.1% vs. 1.8%; *P* = 0.21).

#### Sensitivity analysis

The results of the sensitivity analysis for partial and total tubeless PCNL are presented in Table 4. When the studies of partial tubeless PCNL [11–15, 20–23] were included, most of the outcomes such as hospital stay, return to activities, stone-free rate, postoperative hemoglobin drop, postoperative pain scores, blood transfusion, fever, urine leakage were constant. Though there was no significant difference in operative time between the two groups, it showed a similar tendency across studies. When only the studies of total tubeless PCNL [16–19, 24] were analyzed, only urine leakage was no longer detectable in the sensitivity analysis, the other outcomes remained consistent. Moreover, Ferakis and Stavropoulos [30] have reported that mini-PCNL is usually related to less morbidity than the standard method. A mini-PCNL is defined as a PCNL performed with a sheath diameter ≤ 22F [31]. This mini procedure was used in only two of the included studies [20, 23]. Thus, we performed a sensitivity analysis excluding the mini-PCNL studies. The results show that all outcomes remained consistent (Table 5).

**Table 4:** Results of sensitivity analysis for partial and total tubeless PCNL

Items	No. ofstudies	Reference of studies	Sample size	Tests forheterogeneity		Analysis model	Test for overalleffect		RR/WMD/SMD	Favor
			TB/SD	I^2^	P^a^		Z	P^a^	95% CI	
Analysis of partial tubeless studies										
Stone-free rate	7	[12, 20–23]	234/234	0%	0.830	Fixed	1.22	0.222	1.033[0.980,1.089]	TB
Operative time	5	[11, 12, 14, 20, 23]	118/118	57%	0.054	Random	1.20	0.229^b^	−3.253[−8.547,2.041]	TB
Hospital stay	7	[11–13, 21, 23]	316/316	99%	<0.0001	Random	4.52	<0.0001	−0.931[−1.335,-0.528]	TB
Return to normal activity	4	[11, 13, 15, 21]	226/226	0%	0.528	Fixed	24.76	<0.0001	−3.346[−3.610,-3.081]	TB
Postoperative hemoglobin drop	6	[12, 14, 15, 20, 21, 23]	290/290	48%	0.088	Fixed	1.43	0.152	−0.026[−0.063,0.010]	TB
Postoperative analgesic requirements	6	[11, 12, 14, 15, 21, 23]	286/286	65%	0.013	Random	7.33	<0.0001	−1.262[−1.600,-0.925]	TB
Postoperative pain scores	5	[11, 13–15, 21]	259/258	94%	<0.0001	Random	8.28	<0.0001	−20.750[−25.661,-15.839]	TB
Blood transfusion	4	[11, 13, 14, 22]	90/89	0%	0.761	Fixed	0.63	0.528	1.487[0.434,5.088]	SD
Fever	5	[14, 15, 20–22]	250/248	0%	0.920	Fixed	0.59	0.556	1.253[0.592,2.650]	SD
Urine leakage	4	[14, 15, 20, 21]	233/232	18%	0.303	Fixed	2.45	0.014	0.213[0.062,0.736]	TB
Analysis of total tubeless studies										
Stone-free rate	4	[16, 18, 19, 24]	184/180	0%	0.650	Fixed	0.58	0.565	0.976[0.900,1.059]	SD
Operative time	4	[17, 24]	204/200	55%	0.082	Random	2.00	0.045	−3.844[−7.609,-0.078]	TB
Hospital stay	4	[17, 24]	204/200	98%	<0.0001	Random	2.20	0.028	−1.907[−3.606,-0.207]	TB
Return to normal activity	3	[18, 19, 24]	159/155	99%	<0.0001	Random	1.70	0.090	−5.855[−12.618,0.909]	TB
Postoperative hemoglobin drop	4	[17, 24]	204/200	44%	0.147	Fixed	0.53	0.597	−0.009[−0.043,0.025]	TB
Postoperative analgesic requirements	3	[18, 19, 24]	159/155	97%	<0.0001	Random	1.86	0.062	−1.502[−3.081,0.077]	TB
Postoperative pain scores	2	[19, 24]	124/120	95%	<0.0001	Random	3.01	0.003	−17.448[−28.816,-6.081]	TB
Blood transfusion	3	[17, 18, 24]	136/137	0%	0.746	Fixed	1.29	0.198	0.503[0.176,1.432]	TB
Fever	2	[17, 24]	101/102	0%	0.585	Fixed	0.55	0.583	0.745[0.260,2.133]	TB
Urine leakage	3	[17, 19, 24]	169/165	68%	0.046	Fixed	1.14	0.254^c^	0.351[0.058,2.122]	TB

*TB* Tubeless percutaneous nephrolithotomy, *SD* Standard percutaneous nephrolithotomy, *RR* relative risk, *WMD* Weighted mean difference, *SMD* Standard mean difference, *CI* Confidence interval

^a^P < 0.05 was considered statistically significant

^b^Originally significant before studies using total tubeless percutaneous nephrolithotomy were excluded

^c^Originally significant before studies using partial tubeless percutaneous nephrolithotomy were excluded

**Table 5:** Results of sensitivity analysis for standard PCNL

Items	No. ofstudies	Reference of studies	Sample size	Tests forheterogeneity		Analysis model	Test for overalleffect		RR/WMD/SMD	Favor
			TB/SD	I^2^	P^a^		Z	P^a^	95% CI	
Stone-free rate	10	[12, 18, 19, 21, 22, 24]	463/459	0%	0.910	Fixed	0.32	0.747	0.992 [0.946,1.041]	SD
Operative time	7	[11, 12, 14, 17–19, 24]	266/262	62.8%	0.013	Random	1.98	0.048	−3.436 [−6.837,-0.036]	TB
Hospital stay	10	[11–15, 17–19, 21, 24]	480/476	96.9%	<0.0001	Random	10.02	<0.0001	−1.435 [−1.716,-1.154]	TB
Return to normal activity	7	[11, 13, 15, 18, 19, 21, 24]	385/381	97.5%	<0.0001	Random	5.44	<0.0001	−4.235 [−5.761,-2.709]	TB
Postoperative hemoglobin drop	8	[12, 14, 15, 17–19, 21, 24]	438/434	49.7%	0.053	Fixed	1.37	0.169	−0.018 [−0.043,0.008]	TB
Postoperative analgesic requirements	8	[11, 12, 14, 15, 18, 19, 21, 24]	405/401	91.4%	<0.0001	Random	4.79	<0.0001	−1.234 [−1.739,-0.729]	TB
Postoperative pain scores	7	[11, 13–15, 19, 21, 24]	383/378	94.9%	<0.0001	Random	6.35	<0.0001	−15.787 [−20.660,-10.913]	TB
Blood transfusion	7	[11, 13, 14, 17, 18, 22, 24]	226/226	0%	0.768	Fixed	0.62	0.538	0.785 [0.364,1.696]	TB
Fever	6	[14, 15, 17, 21, 22, 24]	333/333	0%	0.906	Fixed	<0.001	0.999	1.001 [0.521,1.922]	SD
Urine leakage	6	[14, 15, 17, 19, 21, 24]	386/381	40.4%	0.136	Fixed	3.64	<0.0001	0.259[0.125,0.537]	TB

*TB* Tubeless percutaneous nephrolithotomy, *SD* Standard percutaneous nephrolithotomy, *RR* Relative risk; *WMD* weighted mean difference, *SMD* standard mean difference, *CI* confidence interval

^a^P < 0.05 was considered statistically significant

## Discussion

Percutaneous nephrolithotomy is now the major surgical treatment for patients with renal and upper ureteral stones [32, 33]. Using a nephrostomy tube at the end of the PCNL procedure was intended to be an integral part of the procedure [34]; however, it may also cause some significant postoperative discomfort. Therefore, tubeless PCNL has gained widespread popularity in recent years. Patients who were free from nephrostomy tube could be considered as tubeless PCNL.

In this meta-analysis, data from 1148 patients who underwent PCNL from 14 RCTs [11–24] were analyzed to update the effectiveness and safety of tubeless PNCL. Our main findings showed that tubeless PCNL was associated with significantly shorter operative time, shorter hospital stay, and shorter time to return to normal activities. Moreover, lower postoperative pain scores and reduced analgesia requirement were observed in the tubeless PCNL group. There was no significant statistical difference in the postoperative hemoglobin drop or the stone-free rate between the two groups. Concerning the main complications of PCNL, tubeless PCNL could significantly reduce urine leakage. However, no statistically significant difference was found in postoperative fever rate and blood transfusion rate.

The mean operative time was significantly shorter in the tubeless PCNL group than in the standard PCNL group in our analysis. However, in the review by Jiawu Wang et al. [8], there was no statistically significant difference in operative time between the two groups. We considered that these differences in operative times might have been attributed to the study selection. Each study may have calculated operative time using different criteria, and several studies did not provide a clear definition of the operative time involved. Furthermore, patient characteristics and surgeon’s experience were likely the main factors influencing operative time.

The results of this meta-analysis indicated that the hospital stay and the time to return to normal activity were significantly reduced in the tubeless PCNL group. Possible reasons could be attributed to less pain and the procedure did not involve the nephrostomy tube. The use of nephrostomy tubes in the standard PCNL group could have resulted in more postoperative discomfort, as well as requiring an additional procedure for tube removal, which means the prolongation of hospital stay and of time to return to normal activity. Hospital stay plays an important role in the evaluation of tubeless PCNL. Shorter hospital stay and shorter time to return to normal activity could decrease the costs of treatment and improve quality of health-care, which are indicated as advantages of the tubeless procedure.

Tubeless PCNL has been shown to be associated with decreased postoperative pain and analgesic requirements, differences that were statistically significant. Drainage-tube related pain is one of the most common urologic complaints in the standard PCNL patient [35]. Multiple studies have demonstrated significant morbidity associated with indwelling nephrostomy tubes following PCNL, namely increased postoperative discomfort with significant analgesic requirements [14–19, 24, 36]. Thus, by performing tubeless PCNL for selected patients, we can achieve significantly improved patient pain profiles and the restrict usage of analgesic.

We found that the mean postoperative hemoglobin drop was lower in the tubeless PCNL group, but no statistical difference was found between the two groups. Patients with no noticeable hemorrhage during the operation were selected to undergo the tubeless procedure in our study. The main factors influencing the postoperative hemoglobin drop could be postoperative hemorrhage and the use of a nephrostomy tube. In the study by Shoma et al. [37], placement of a nephrostomy tube did not decrease the postoperative hemoglobin drop nor the development of perinephric hematoma. Furthermore, several investigators [14, 15, 17–21, 23] reported there was no significant difference between the two groups. The change in hemoglobin level may not be related to the use of a nephrostomy tube.

In our meta-analysis, there was no significant difference between the two groups in the stone-free rate; which is also similar to results of previous reviews [7, 9] and other published studies [11–24, 38]. The nephrostomy tube was placed at the end of the procedure in the standard group without affecting the stone-free rate. The incidence of stone clearance may be associated with the stone characteristics and renal anatomy in selected patients [24]. In addition, we found the vast majority of the included studies [12–14, 16, 18–22, 24] could not achieve a stone clearance of 100%. Although there were strict inclusion criteria, four studies [13, 18, 21, 22] in our review still reported the requirement of a second PCNL to treat the residual stone. It is sometimes difficult to exclude the existence of residual stones at the completion of the procedure. In some patients, performing a new puncture tract may sometimes be unavoidable [39]. This could represent a potential limitation of the tubeless procedure. However, flexible ureteroscopy has become a viable option for the treatment of renal stones in recent years due to its high stone-free rate and low morbidity [40]. Thus, it may be considered as a more suitable alternative to manage the residual stone.

The standard approach to PCNL includes placement of a nephrostomy tube designed to aid in hemostasis and drain the pelvicalyceal system [37]. However, our meta-analysis indicated that the tubeless PCNL did result in any increase in related complications. There was no significant difference in postoperative fever and blood transfusion between the two groups. Moreover, tubeless PCNL could significantly diminish urine leakage in comparison to the standard PCNL group. In our review, standard PCNL was not superior in terms of postoperative complications. Of course, this may be due to the fact that the study consisted of strictly selected patients with no complete or partial staghorn calculi, no congenital urinary tract anomalies, no noticeable hemorrhage during the operation, and no major collecting-system injury. In addition, many innovative techniques have been used recently to prevent postoperative bleeding and urinary leakage in the absence of an indwelling nephrostomy tube [41]. Santosh Kumar et al. [24] occluded the access tract with a ‘Santosh-PGI hemostatic seal’ following the stone clearance in the tubeless group and Shah et al. [42] instilled a fibrin sealant and gelatin matrix hemostatic sealant in the percutaneous tract after the completion of PCNL. Of course, the use of sealants remains controversial. Whether or not sealants can decrease bleeding and urinary extravasation deserves further exploration [43]. Nonetheless, tubeless PCNL may still result in fewer complications in appropriately selected patients when compared to PCNL performed with the presence of nephrostomy tube.

Though we have described many advantages of tubeless PCNL, it still has some limitations. One important issue pertinent to partial tubeless PCNL is the complication of the indwelling ureteral stent. Limb and Bellman [44] have reported that a subset of young men was unable to tolerate the internal Double-J stent. An additional drawback of tubeless PCNL is that it may interfere with a subsequent routine second-look procedure required to clear the residual stone fragments. In the study of Singh et al. [13], three patients had missed residual stones (invisible on initial postoperative fluoroscopy) that become apparent later. Last but not the least, tubeless PCNL may only be suitable for carefully selected patients.

Patients with ureteric obstruction, significant bleeding during the surgery, major collecting-system injury, or abnormal renal function may require a nephrostomy tube to aid in hemostasis and to drain the pelvicalyceal system. Patients without the above conditions were included in the study. We excluded those patients who underwent bilateral PCNL simultaneously. Though a small series of patients treated with simultaneous bilateral tubeless PNL have been reported [45, 46], and a timely drainage of the kidneys to prevent renal function damage should be considered. Furthermore, patients with staghorn stones, congenital urinary tract anomalies, serious urinary infection, solitary functioning kidneys, or kidneys with prior open surgery were also excluded from the study. Patients with staghorn stones always required subsequent surgery, and removing percutaneous access by the tubeless procedure may have necessitated a repeat of the PNL [43]. Patients with congenital urinary tract anomalies, serious urinary infection, and solitary functioning kidneys also need timely renal drainage which means they were unfit for tubeless PCNL. Finally, since prior open renal surgery might cause ureteral damage, we did not consider these patients in our study.

Some potential limitations of our meta-analysis should be considered. Firstly, we did not define the specific size of the stone as inclusion criteria due to the lack standards in some studies, however all patients included in the studies were suited to PCNL. Secondly, we did not analyze other potential complications, except for those mentioned above, such as pleural effusion, postoperative urinary tract infection, and septicemia. Due to the lack of adequate relevant data, these complications could not be included in our meta-analysis. Thirdly, the studies did not unify the category and specifications of postoperative analgesia, which may have led to a potential bias. We hope that uniform treatment standards will be discussed and defined in the future. Fourthly, due to the lack of sufficient description in some studies or in the limitations in the design and implementation of the included studies, the assessment of article quality could not be performed. This may have resulted in a potential bias. Finally, the pooled results relative to operative time, hospital stay, postoperative pain scores, and the time to return to normal activities showed significant heterogeneity. Thus, subgroup analysis, influence analysis, and sensitivity analysis were all performed to reduce heterogeneity, but these analyses did not significantly alter the results. Consequently, statistical heterogeneity may have influenced the conclusions. Lastly, the Begg’s test of postoperative pain scores indicated that there might have been a publication bias for the included studies.

## Conclusions

In conclusion, this updated meta-analysis showed that tubeless PCNL may be an effective and safe procedure for selected patients, resulting in significantly shorter hospital stay and shorter time to return to normal activity. Moreover, lower postoperative pain scores, reduced analgesia requirement, and urine leakage were also observed in tubeless PCNL without increasing other complications. We consider tubeless PCNL an acceptable and safe management option with experience and careful patient selection. Of course, larger high quality multi-center long-term RCTs are required to confirm the outcomes of our meta-analysis in the future.

## Abbreviations

BMI: Body mass Index
CI: Confidence interval
PCNL: Percutaneous nephrolithotomy
POD: Postoperative day
RCTs: Randomized controlled trials
RR: Relative risk
SD: Standard deviation
SMD: Standardized mean difference
UTI: Urinary tract infection
VAS: Visual analog scale
WMD: Weighted mean difference

## References

[1] Türk C, Neisius A, Petrik A, et al. Guidelines on urolithiasis. EAU. 2017. Available at: http://uroweb.org/guideline/urolithiasis/. Accessed 15 April 2017.

[2] Geraghty R, Jones P, Somani BK (2017). Worldwide trends of urinary stone disease treatment over the last two decades: a systematic review. J Endourol.

[3] Ganpule AP, Vijayakumar M, Malpani A (2016). Percutaneous nephrolithotomy (PCNL) a critical review. Int J Surg.

[4] Tirtayasa PMW, Yuri P, Birowo P, et al. Safety of tubeless or totally tubeless drainage and nephrostomy tube as a drainage following percutaneous nephrolithotomy: A comprehensive review. Asian J Surg. 2016;doi: 10.1016/j.asjsur.2016.03.003. 10.1016/j.asjsur.2016.03.003 27235306

[5] Bellman GC, Davidoff R, Candela J (1997). Tubeless percutaneous renal surgery. J Urol.

[6] Song G, Guo X, Niu G (2015). Advantages of tubeless mini-percutaneous nephrolithotomy in the treatment of preschool children under 3 years old. J Pediatr Surg.

[7] Yuan H, Zheng S, Liu L (2011). The efficacy and safety of tubeless percutaneous nephrolithotomy: a systematic review and meta-analysis. Urol Res.

[8] Wang J, Zhao C, Zhang C (2012). Tubeless vs standard percutaneous nephrolithotomy: a meta-analysis. BJU Int.

[9] Zhong Q, Zheng C, Mo J (2013). Total tubeless versus standard percutaneous nephrolithotomy: a meta-analysis. J Endourol.

[10] Mandhani A, Goyal R, Vijjan V (2007). Tubeless percutaneous nephrolithotomy -- should a stent be an integral part?. J Urol.

[11] Choi M, Brusky J, Weaver J, et al. Randomized trial comparing modified tubeless percutaneous nephrolithotomy with tailed stent with percutaneous nephrostomy with small-bore tube. J Endourol. 2006;20(10):766-70. 10.1089/end.2006.20.766 17094752

[12] Tefekli A, Altunrende F, Tepeler K (2007). Tubeless percutaneous nephrolithotomy in selected patients: a prospective randomized comparison. Int Urol Nephrol.

[13] Singh I, Singh A, Mittal G (2008). Tubeless percutaneous nephrolithotomy: is it really less morbid?. J Endourol.

[14] Shah HN, Sodha HS, Khandkar AA (2008). A randomized trial evaluating type of nephrostomy drainage after percutaneous nephrolithotomy: small bore v tubeless. J Endourol.

[15] Agrawal MS, Agrawal M, Gupta A (2008). A randomized comparison of tubeless and standard percutaneous nephrolithotomy. J Endourol.

[16] Crook TJ, Lockyer CR, Keoghane SR (2008). A randomized controlled trial of nephrostomy placement versus tubeless percutaneous nephrolithotomy. J Urol.

[17] Istanbulluoglu MO, Ozturk B, Gonen M (2009). Effectiveness of totally tubeless percutaneous nephrolithotomy in selected patients: a prospective randomized study. Int Urol Nephrol.

[18] Aghamir SMK, Modaresi SS, Aloosh M (2011). Totally tubeless percutaneous nephrolithotomy for upper pole renal stone using subcostal access. J Endourol.

[19] Chang CH, Wang CJ, Huang SW (2011). Totally tubeless percutaneous nephrolithotomy: a prospective randomized controlled study. Urol Res.

[20] Lu Y, Ping JG, Zhao XJ (2013). Randomized prospective trial of tubeless versus conventional minimally invasive percutaneous nephrolithotomy. World J Urol.

[21] Agrawal MS, Sharma M, Agarwal K (2014). Tubeless percutaneous Nephrolithotomy using Antegrade tether: a randomized study. J Endourol.

[22] Zhao PT, Hoenig DM, Smith AD (2016). A randomized controlled comparison of nephrostomy drainage vs ureteral stent following percutaneous nephrolithotomy using the Wisconsin stone QOL. J Endourol.

[23] Sebaey A, Khalil MM, Soliman T (2016). Standard versus tubeless mini-percutaneous nephrolithotomy: a randomised controlled trial. Arab journal of urology.

[24] Kumar S, Singh S, Singh P (2016). Day care PNL using 'Santosh-PGI hemostatic seal' versus standard PNL: a randomized controlled study. Cent European J Urol.

[25] Moher D, Liberati A, Tetzlaff J. Preferred reporting items for systematic reviews and meta-analyses: the PRISMA statement. BMJ. 2009; 10.1136/bmj.b2535. 10.1136/bmj.b2535 PMC2714657 19622551

[26] Huskisson EC (1974). Measurement of pain. Lancet.

[27] Phillips B, Ball C, Sackett D, et al. Oxford Centre for Evidence-Based Medicine Levels of Evidence. CEBM. 2017. http://www.cebm.net/ocebm-levels-of-evidence/. Accessed 15 Apr 2017.

[28] Higgins JPT, Green S. Cochrane handbook for systematic reviews of interventions Version 5.1.0 [updated June 2017]. The Cocharane Collaboration 2017. http://handbook.cochrane.org. Accessed 15 Apr 2017.

[29] Hozo SP, Djulbegovic B, Hozo I, et al. Estimating the mean and variance from the median, range, and the size of a sample. BMC Med Res Methodol. 2005; 10.1186/1471-2288-5-13. 10.1186/1471-2288-5-13 PMC1097734 15840177

[30] Ferakis N, Stavropoulos M (2015). Mini percutaneous nephrolithotomy in the treatment of renal and upper ureteral stones: lessons learned from a review of the literature. Urology annals..

[31] Schilling D, Husch T, Bader M (2015). Nomenclature in PCNL or the tower of babel: a proposal for a uniform terminology. World J Urol.

[32] Segura JW, Patterson DE, LeRoy AJ (1985). Percutaneous removal of kidney stones: review of 1,000 cases. J Urol.

[33] Skolarikos A, Alivizatos G, de la Rosette JJMC (2005). Percutaneous nephrolithotomy and its legacy. Eur Urol..

[34] Maheshwari PN, Andankar MG, Bansal M (2000). Nephrostomy tube after percutaneous nephrolithotomy: large-bore or pigtail catheter?. J Endourol.

[35] Abbott JE, Deem SG, Mosley N (2016). Are we fearful of tubeless percutaneous nephrolithotomy? Assessing the need for tube drainage following percutaneous nephrolithotomy. Urology annals.

[36] Pietrow PK, Auge BK, Lallas CD (2003). Pain after percutaneous nephrolithotomy: impact of nephrostomy tube size. J Endourol.

[37] Shoma AM, Elshal AM (2012). Nephrostomy tube placement after percutaneous nephrolithotomy: critical evaluation through a prospective randomized study. Urology.

[38] Ni S, Qiyin C, Tao W (2011). Tubeless percutaneous nephrolithotomy is associated with less pain and shorter hospitalization compared with standard or small bore drainage: a meta-analysis of randomized controlled trials. Urology.

[39] Kara C, Resorlu B, Bayindir M (2010). A randomized comparison of totally tubeless and standard percutaneous nephrolithotomy in elderly patients. Urology.

[40] Proietti S, Knoll T, Giusti G (2016). Contemporary ureteroscopic management of renal stones. Int J Surg.

[41] de Cogain MR, Krambeck AE (2013). Advances in tubeless percutaneous nephrolithotomy and patient selection: an update. Current urology reports.

[42] Shah HN, Hegde S, Shah JN (2006). A prospective, randomized trial evaluating the safety and efficacy of fibrin sealant in tubeless percutaneous nephrolithotomy. J Urol.

[43] Zilberman DE, Lipkin ME, de la Rosette JJ (2010). Tubeless percutaneous nephrolithotomy--the new standard of care?. J Urol.

[44] Limb J, Bellman GC (2002). Tubeless percutaneous renal surgery: review of first 112 patients. Urology.

[45] Istanbulluoglu MO, Ozturk B, Cicek T (2009). Bilateral simultaneous totally tubeless percutaneous nephrolithotomy: preliminary report of six cases. J Endourol.

[46] Bagrodia A, Raman JD, Bensalah K (2009). Synchronous bilateral percutaneous nephrostolithotomy: analysis of clinical outcomes, cost and surgeon reimbursement. J Urol.

